# Prediction of "hot spots" of aggregation in disease-linked polypeptides

**DOI:** 10.1186/1472-6807-5-18

**Published:** 2005-09-30

**Authors:** Natalia Sánchez de Groot, Irantzu Pallarés, Francesc X Avilés, Josep Vendrell, Salvador Ventura

**Affiliations:** 1 Departament de Bioquímica i Biologia Molecular, Facultat de Ciències, Universitat Autònoma de Barcelona, E-08193 Bellaterra, Spain; 2 Institut de Biotecnologia i de Biomedicina, Universitat Autònoma de Barcelona, E-08193 Bellaterra, Spain

## Abstract

**Background:**

The polypeptides involved in amyloidogenesis may be globular proteins with a defined 3D-structure or natively unfolded proteins. The first class includes polypeptides such as β2-microglobulin, lysozyme, transthyretin or the prion protein, whereas β-amyloid peptide, amylin or α-synuclein all belong to the second class. Recent studies suggest that specific regions in the proteins act as "hot spots" driving aggregation. This should be especially relevant for natively unfolded proteins or unfolded states of globular proteins as they lack significant secondary and tertiary structure and specific intra-chain interactions that can mask these aggregation-prone regions. Prediction of such sequence stretches is important since they are potential therapeutic targets.

**Results:**

In this study we exploited the experimental data obtained in an *in vivo *system using β-amyloid peptide as a model to derive the individual aggregation propensities of natural amino acids. These data are used to generate aggregation profiles for different disease-related polypeptides. The approach detects the presence of "hot spots" which have been already validated experimentally in the literature and provides insights into the effect of disease-linked mutations in these polypeptides.

**Conclusion:**

The proposed method might become a useful tool for the future development of sequence-targeted anti-aggregation pharmaceuticals.

## Background

In the last decade, protein aggregation has moved beyond being a mostly ignored area of protein chemistry to become a key topic in medical sciences [1], mainly because the presence of insoluble deposits in human tissues correlates with the development of many debilitating human disorders including the amyloidoses and several neurodegenerative diseases [2]. The proteins involved in these diseases are not related in terms of sequence or secondary structure content. From the conformational point of view, two major classes can be distinguished: globular proteins with a stable unique conformation in the native state and intrinsically unstructured proteins [3]. Globular proteins rarely aggregate from their native states and destabilization, resulting in an increased population of unfolded molecules, is well established as a trigging factor in disorders associated with the deposition of proteins that are globular in their normal functional states [4], as in the cases of β2-microglobulin, lysozyme, transthyretin and the prion protein. Interestingly enough, many proteins involved in depositional disorders are mostly unstructured within the cell [3]. These include amylin, amyloid-β-protein, and α-synuclein, among others. In these cases, protein deposition does not require unfolding and can occur by direct self-assembly of the unstructured polypeptide chains.

One of the major unanswered questions of protein aggregation is the specificity with which the primary sequence determines the aggregation propensity from totally or partially unfolded states. Deciphering the answer to this question will give us a chance to control the unwanted protein deposition events through specific sequence-targeted therapeutics. A first advance in this direction is the recent discovery that not all regions of a polypeptide are equally important for determining its aggregation tendency, both in natively unfolded and globular proteins. In this way, some authors, including ourselves, have proved recently that very short specific amino acid stretches can act as facilitators or inhibitors of amyloid fibril formation [5,6]. These relevant regions are usually known as aggregation "hot spots". Aggregation-prone regions are likely to be blocked in the native state of globular proteins because their side chains are usually hidden in the inner hydrophobic core or already involved in the network of contacts that stabilizes a protein. This accounts for the protective role of the native structure against aggregation [7]. In contrast, aggregation-prone regions are already exposed to solvent in natively unfolded proteins, available for the establishment of inter-molecular contacts that may finally lead to the formation of aggregates. Accordingly, the presence of putative "hot spots" of aggregation is much more frequent in the sequences of globular proteins than in those coding for natively unfolded proteins [8]. The presence of aggregation-prone regions has been described in most of the peptides and proteins underlying neurodegenerative and systemic amyloidogenic disorders [9].

We have used a simple *in vivo *system to study the aggregation effects of a complete set of mutations in one of the best characterized "hot spots" in a disease-linked protein: the central hydrophobic cluster (CHC) of the Amyloid-β-protein (Aβ) [10,11]. The results in this and other studies on protein models not related to disease [12], suggested that common and simple principles underlie protein aggregation, at least from totally or partially unfolded states, and that the propensities of proteins backbones to aggregate are sharply modulated by the sequences that dress them. Based on these assumptions, we have developed a simple approach that identifies the presence of "hot-spots" of aggregation in globular and unstructured disease-linked polypeptides and predicts the aggregation effects of mutations in their sequences.

## Results and discussion

### Aggregation propensities of natural amino acids

The rationale behind our study is based on two recent observations in the field. First, not all the polypeptide sequence is relevant for the aggregation of a given protein, but rather there exist specific regions that drive the process [5,6] and second, similar simple rules appear to underlie the aggregation propensities of unrelated proteins from unfolded states [12]. According to these two assumptions one may expect that the conclusions obtained from the study of a relevant "hot spot" of aggregation in a specific protein could apply to other unrelated proteins involved in disease. As commented upon previously, we have exploited an *in vivo *reporter method to calculate the relative aggregation propensities of each individual natural amino acid when placed in the central position of the CHC of Aβ (see Material and Methods). The highest aggregation propensities correspond to isoleucine, phenylalanine, valine, and leucine, whereas aspartic, glutamic, asparagine, and arginine exhibit the lowest (Table 1). In general, hydrophobic residues tend to induce aggregation whereas polar ones promote solubility, matching the general assumption that hydrophobic interactions are supposed to play an important role in protein aggregation [13].

### Generation of protein aggregation profiles and prediction of the effects of protein mutation on the aggregation propensity

Provided that a given polypeptide aggregates from an at least partially unstructured state, the experimental intrinsic aggregation propensities shown in Table 1 should apply independently of the protein context. Thus, a profile can be theoretically generated for any protein sequence to detect those regions with aggregation propensities above the average value of the whole sequence. This leads directly to the definition of "hot spot" of aggregation as a certain region that displays higher aggregation propensity than the rest of the sequence. Interestingly, a related approach has been reported very recently for the analysis of unstructured proteins associated with neurodegenerative diseases[14].

A good number of natural occurring mutations have been reported in proteins associated to depositional diseases. In many cases they result in changes in the global protein aggregation propensity and sometimes in the appearance of premature or acute pathological symptoms. The change in average aggregation propensity (ΔAP) between the wild type and the different mutants should predict the effect of sequence variations on the aggregation propensities, provided that they rely on changes in the intrinsic polypeptide properties.

### Analysis of disease-related polypeptide sequences

In this section the above described analysis is applied to a set of proteins linked to depositional diseases and the obtained results are compared with the available experimental data.

### Intrinsically unstructured proteins

#### Amyloid-β-protein

**Figure 1:**
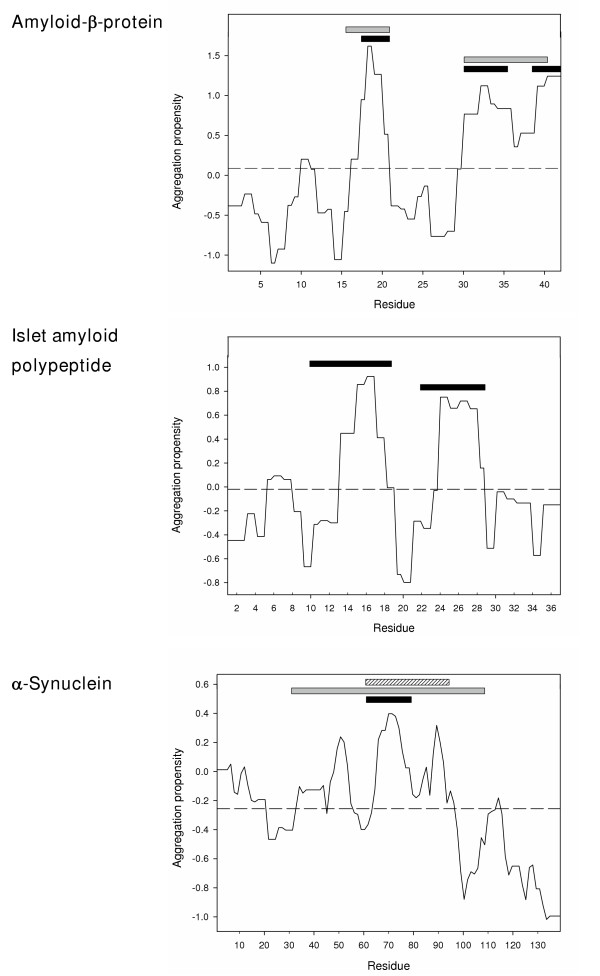
**Aggregation profile of natively unfolded proteins related to disease**. The average aggregation propensity of the different polypeptides is shown as a dashed line. Minimal protein regions which have been experimentally proven to be involved in aggregation are shown at the top of the plot as black bars. Regions in the core of the fibrils are shown as grey bars (when information available). The NAC fragment of α-Synuclein is shown as a dashed bar.

As a proof of principle our approach was first tested in the molecule from which the experimental amino acid aggregation propensities were derived. Alzheimer's disease (AD) is a progressive neurodegenerative disorder characterized by the patient's memory loss and impairment of cognitive abilities. The extracellular amyloid is found in the brain and is widely believed to be involved in the progression of the disease [15]. The principal component of the lesions is the hydrophobic polypeptide Aβ. The most abundant forms found in amyloid plaques are a 40-mer (Aβ40) and a 42-mer (Aβ42). Although less abundant, Aβ42 is more amyloidogenic than Aβ40 and is the major component of neuritic plaques [16]. Two main regions with high aggregation propensity can be distinguished in the aggregation profile for this polypeptide (Fig. 1). The second region arises from the contribution of two sequence stretches comprising residues 30–36 and 38–42, respectively. The predicted aggregation-prone regions are in excellent agreement with the experimental data in the literature. Residues 16–21 overlap with the CHC sequence comprising residues 17–21, a particular region recognized to play a key role in Aβ aggregation and that is defined as specially relevant for the amyloidogenesis of the Aβ40 and Aβ42 peptides by two recent proline-scanning-mutagenesis studies [17,18]. In addition, structural studies using solid state-NMR [19] and site-directed spin labeling [20] have revealed that residues 16–21 are located in the core of Aβ fibrils. Accordingly, a short 7 residues fragment comprising residues 16–22 is able to form ordered amyloid fibrils [21] and, more interestingly, 16-LVAFF-20 and derived peptides have been shown to bind to Aβ42 and act as potent inhibitors of amyloid formation [22]. The region 30–42, including both 30–36 and 38–42 stretches, has been also implicated in Aβ aggregation. Proline-scanning-mutagenesis revealed that the region 31–36 is sensitive to proline replacement and likely to include a β-sheet portion of the Aβ fibrils [17,18]. The contribution of the C-terminal region 38–42 to Aβ amyloidogenesis becomes clear from the observation that, although Aβ40 is produced in greater abundance *in vivo*, the prevalence of the full-length 42-mer in plaques is much higher [16]. Experiments with truncated synthetic Aβ peptides have confirmed that Aβ39 and Aβ40 are kinetically soluble for several days, whereas Aβ42 immediately aggregates into amyloid fibrils [23]. The relevance of the predicted 30–42 region is confirmed by structural studies that demonstrate that residues 30–40 are located in the core of the Aβ fibrils [20].

A set of mutations in the CHC and adjacent positions of Aβ42 is intimately associated to early-onset familial Alzheimer diseases (FAD). The substitutions include A21G (Flemish), E22Q (Dutch) and E22G (Arctic) [24]. Aβ42 congeners bearing these mutations display distinct aggregation kinetics. The rate of fibril formation by the Flemish mutant is decreased relative to WT Aβ42, whereas the Dutch mutant peptide aggregates substantially faster. The Arctic peptide does not shows an overall change in the rate of fibrillogenesis relative to WT Aβ42, but rather accelerated protofibril formation. To assess whether the effect of such mutations could be predicted by the present approach we calculated ΔAP for the different sequences. The results obtained describe accurately the effects documented in the literature (Table 2).

Adding to the mutations present in the population, a large set of mutations has been artificially introduced on Aβ that result in changes in its aggregation propensity. ΔAP values were also calculated for several of them and the results compared with the experimental data (Table 2). The calculated changes in aggregation propensity are in excellent agreement with the trends reported in the literature. Briefly, we predict the changes in aggregation of F19 mutants, those of I31 and I32 in the 30–36 region and those of I41 and A42 in the C-terminal region, as well as the effects of deletions both in the N and the C ends. Finally, we also predict the high solubility of Aβ versions generated by random mutagenesis [25].

#### Islet amyloid polypeptide

Type II diabetes is associated with progressive beta-cell failure manifested as a decline in insulin secretion and increasing hyperglycemia. A growing body of evidence suggests that beta-cell failure in type II diabetes correlates with the formation of pancreatic islet amyloid. Islet amyloid polypeptide (IAPP, amylin), the major component of islet amyloid, is co-secreted with insulin from beta-cells. In type II diabetes, this peptide aggregates to form amyloid fibrils that are toxic to beta-cells [26]. IAPP is an unstructured peptide hormone of 37 amino acid residues. Two "hot spots" of aggregation comprising residues 12–18 and 22–28 are detected for this peptide (Fig. 1). Interestingly enough, a 8–37 IAPP-fragment including both "hot spots", has been shown to form amyloid fibrils under physiological conditions [27]. The two aggregation prone regions sharply coincide with those protected in the core of the fibrils in a recently described structural model of IAPP aggregates [28]. In this study, residues 12–17 and 22–27 are proposed to form the inner β-sheets in the fibril protofilament structure. According to this hypothesis, peptides corresponding to residues 8–20, 10–19, 20–29 of human IAPP, which include one of the "hot spots" described here, all form amyloid [29-31]. Smaller peptides derived from these regions have also been shown to form amyloid, and a recent investigation suggests that the minimal amyloid forming fragment of IAPP consists of residues 22–27. This hexapeptide fragment, NFGAIL, forms β-sheet-containing fibrils that coil around each other in typical amyloid fibril morphology [32].

The analysis also explains the available mutational data on IAPP. Diabetes-associated IAPP amyloid occurs in primates and cats but not in rodents [33]. Consistently, the sequences of peptides 20–29 of rodents display reduced average aggregation propensity relative to that of cat and human (Table 3). We also predict the slightly increased aggregation propensities of single or multiple mutations of rat IAPP to the corresponding residues of human IAPP [33]: R18H, L23F or V26I, as well as the results from alanine-scanning-mutagenesis in a peptide encompassing residues 22–27 [32] (Table 3). It has been found that a substitution at position 20 (S20G) in the IAPP molecule in a reduced subpopulation of Japanese people with type II diabetes is associated with an earlier onset and more severe form of disease [34]. In this case, our approach does no predict an increased but a slightly reduced aggregation propensity in the mutant, suggesting that the pathological symptoms in this variant may arise from non-intrinsic factors. In fact, it has been suggested that the accelerated aggregation of the S20G variant could be related to structural reasons, resulting from a better packing of the turns connecting the β-sheets in the final protofilament structure [28] that cannot be predicted by the present approach.

Several mechanisms have been proposed for IAPP fibril formation in type II diabetes. One widely accepted mechanism is that in type II diabetes, increased production and secretion of IAPP associated with increased demand for insulin might result in accumulation and aggregation of IAPP [35]. A second view considers that impaired processing of the IAPP precursor molecule, proIAPP, by islet beta-cells may lead to hypersecretion of unprocessed or partially processed forms of proIAPP that may have a higher tendency for aggregation compared to mature IAPP [35]. Our calculated average aggregation propensities for proIAPP and processed IAPP support this view (Table 3).

#### α-Synuclein

Parkinson disease is the most common neurodegenerative movement disorder and is pathologically characterized by the presence of neuronal intracytoplasmatic deposits of aggregated protein called Lewy bodies [36]. Lewy bodies also occur in other cognitive disorders, globally known as α-synucleinopathies. α-Synuclein is the major component of the fibrils that form the Lewy bodies [36]. It is a small (137 residues), natively unfolded, soluble, presynaptic and highly conserved protein without a well-defined function. The aggregation profile for this polypeptide is shown in Fig. 1. Several large aggregation-prone stretches were predicted for the α-Synuclein sequence: region 1–18, region 27–56 and specially region 61–94. Again, our predictions are in complete agreement with the experimental data in the literature, as many studies suggest that the central region of the protein, known as the non-Aβ component of amyloid plaques (NAC, amino acids 61–95), is the responsible for its aggregation process [37]. A peptide comprising residues 68–78 of α-synuclein has been shown to be the minimum fragment that, like α-synuclein itself, forms amyloid fibrils and exhibits toxicity towards cells in culture [38]. This fragment is included in the region 62–80 which we predict as the sequence stretch with the highest aggregation propensity. All the α-synucleinopathies are characterized by the accumulation of the 35 residues NAC fragment in the insoluble deposits [37]. Accordingly, this central region is predicted to have a much higher average aggregation propensity than its soluble precursor (ΔAP = +38.47). The importance of this hydrophobic stretch is further supported by its absence in β-synuclein, a homologue of α-synuclein, with strongly reduced propensity for fibril formation. It has been shown that the deletion of amino acids 71–82 within the hydrophobic region abrogated the ability of human α-synuclein to polymerize into fibrils [39]. Protease digestion studies suggest that the core region of α-synuclein in the fibrils could be longer, since a 7-kDa fragment (comprising residues 31–109) was shown to be protected from proteinase K digestion [40]. This region contains the putative 12-residue core domain, as well as the NAC region and includes the second and third "hot spots" in our profile. A structural study on the organization of α-synuclein in the fibrilar state using site-directed spin labelling confirms that the 34–101 residues region constitutes the core of the fibrils forming a parallel in-register β-sheet structure whereas the N terminus is structurally more heterogeneous and the C terminus (40 amino acids) is completely unfolded [41].

Several α-Synuclein mutations appear associated with familial early-onset Parkinson Disease: A30P, A53T and E46K. All they map into our predicted second "hot spot". The rates of fibril assembly of the E46K and A53T mutants have been shown to be greater than those of the wild type and A30P proteins [42]. We predict a similar average aggregation propensity for the wild-type and the A30P mutant and an slightly increased aggregation propensity for the E46K mutant, but fail to foresee the effect of the A53T mutation in promoting the formation of protofibrils. Obviously, other functional factors apart from the intrinsic aggregation propensities can strongly influence the aggregation tendency of unfolded polypeptide chains within the cell. In fact the effects of α-synuclein mutations have been associated either to an impaired degradation inside lysosomes or to a reduced axonal transport of the variants [43,44]. Both situations may result in increased concentrations of the protein in certain regions of the neuron that may favor the nucleation step of amyloid formation. According to this, α-synuclein gene triplication identified in two independent families [45] has been shown to accelerate the development of Parkinson disease. Thus, an increase in the amount of cellular α-synuclein appears to be important for the pathogenesis of Parkinson disease, suggesting that the effects of the different α-synuclein mutations on protein aggregation could be quantitative, in terms of local concentration, rather that qualitative. Thus, experimental deviations from the theoretical predictions in natively unfolded proteins, in addition to reflect limitations of the approach, might also contain relevant information, prompting to find alternative structural, as in the case of amylin, or functional, as in the case of α-synuclein, explanations for the observed behavior.

### Globular proteins

#### β2-Microglobulin

**Figure 2:**
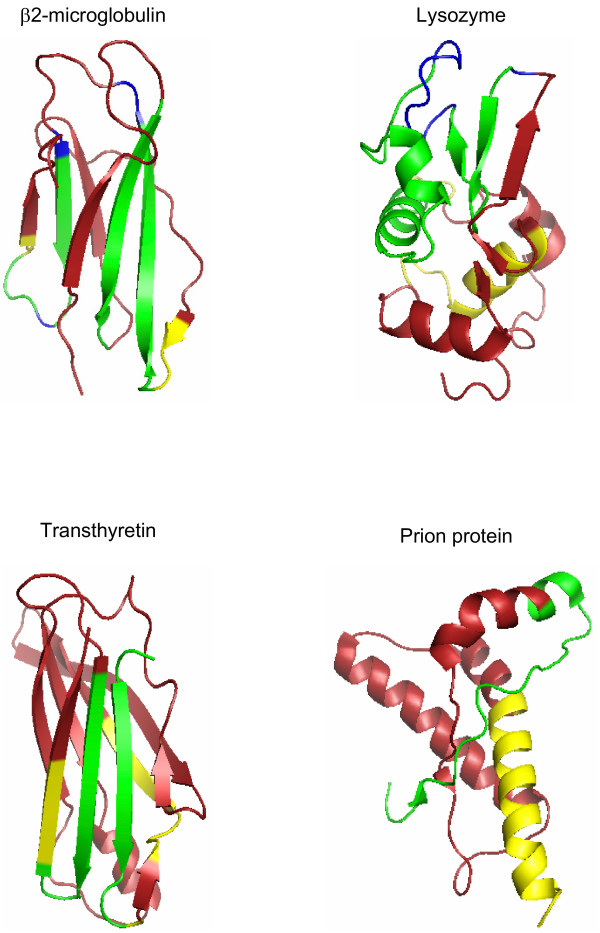
**Representation of the 3D structure of globular proteins related to disease**. The chain segments in which the prediction and the experimental data coincide are colored in green. Those identified experimentally to be relevant for amyloid formation but not predicted by the present approach are colored in blue. The regions predicted to be important for amyloid formation from which experimental data are not available or indicates that they are not involved in aggregation are shown in yellow.

**Figure 3:**
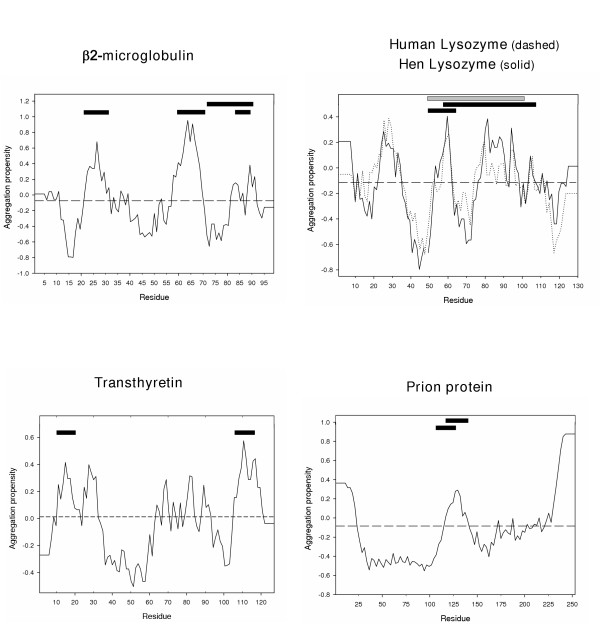
**Aggregation profile of globular proteins related to disease**. Minimal protein regions which have been proved experimentally to be involved in aggregation are shown at the top of the plot as black bars. Regions in the core of the fibrils are shown as a grey bars (when information available).

β2-Microglobulin-related amyloidosis is a common and serious complication in patients on longterm hemodialysis [46]. Intact β2-microglobulin is a major structural component of the amyloid fibrils. β2-Microglobulin (β2-m) is a small (99 residues) non-glycosilated protein with an immunoglobulin-like fold consisting in two antiparallel pleated β-sheets linked by a disulfide bond (Fig. 2). β2-m has been shown to form amyloid fibrils *in vitro *under different conditions, but in all cases β2-m populates unfolded non-native states as precursors to fibril assembly [47]. Under these conditions aggregation-prone regions, if present, may promote and drive the aggregation event. According to the analysis of the aggregation profile, shown in Fig. 3, this protein displays four "hot spots" encompassing residues 21–31, 56–69 and 79–85, and 87–91. These regions sharply coincide with four different secondary structure elements in β2-m: β-strand 2, formed by residues 21–31; β-strand 6, formed by residues 61–71; β-strand 7; formed by residues 77–85 and β-strand 8, formed by residues 86–95 (Fig. 2). In agreement with our prediction a peptide comprising residues 21–41 has been shown to form fibrils in isolation [48]. In addition, a N-terminal fragment of this short peptide corresponding exactly to our first "hot spot" [21-31] is also able to self-assemble into fibrillar structures [49]. Interestingly enough, the peptides 23–31 and 21–29 exhibited reduced amyloidogenesis [49]. Thus, in this particular "hot spot" the prediction delimits not only the overall region important for aggregation but also its precise size. The amino acid stretches 59–79 and its shorter version 59–71 which overlap with the predicted second aggregation-prone region of β2-m have been also shown to form fibrils [50]. The C-terminal fragment 72–99 of β2-m has been also reported to form amyloid [51]. This 29 residues sequence includes our third and fourth "hot spots" of aggregation. The peptide 91–99 does not aggregate, indicating that the last 9 residues of β2-m are not relevant for amyloidogenesis as predicted here [49]. The N-terminal region, for which no aggregation propensity is predicted, is probably not involved inthe aggregation process as evidenced by the fact that the fragment 6–12 does not form fibrils [49]. This observation could be physiologically relevant since the N-terminus of β2-m is truncated in 30% of the molecules extracted from ex vivo fibrils [52].

In contrast to the human protein, mouse β2-m does not form fibrils even at high concentration [53]. Based on this observation a seven residues region corresponding to residues 83–89 of human β2-m has been suggested to be particularly important for aggregation, since it corresponds to the sequence with the highest divergence between both species. This hypothesis has been tested experimentally, since a heptapeptide bearing the human sequence is able to self-assemble whereas the mouse version is not [53]. The complete mouse sequence is predicted to have a strongly reduced aggregation propensity (ΔAP = -47.86).

Overall, our predictions on the presence and location of "hot spots" in β2-m are extremely accurate and overlap with the experimentally found relevant regions (Fig. 2). The observation that short peptides including the aggregation-prone regions described here form amyloids implies that exposure of previously hidden short segments can nucleate native proteins into the amyloid state and reinforces the hypothesis that fibril formation is sequence specific.

One of the most urgent issues in the study of amyloid fibrils is to reproduce the formation of fibrils under physiological conditions. Recently, it has been found that low concentrations of SDS around the critical micelle concentration induce the extensive growth of β2-m amyloid fibrils at physiological pH, probably through the SDS-induced conformational change of β2-m monomers [54]. Contrarily to what was expected, the presence of low concentration of SDS had little effect on the stability of the protein and did not promote global protein unfolding. Our results strongly suggest that in β2-m the parts of the molecule involved in aggregation are located in pre-formed β-strands. Therefore, it is possible that local unfolding events may allow anomalous intermolecular interaction between this preformed elements leading to the formation of an aggregated β-sheet structure. This would explain the formation of amyloid deposits in hemodyalisis patients in which no major unfolding of the protein is expected to occur, as well as the effect of seeds, which may have exposed aggregation prone β-strands, in strongly accelerating the aggregation process of β2-m under physiological conditions [55].

#### Lysozyme

Human lysozyme has been shown to form amyloid fibrils in individuals suffering from nonneuropathic systemic amyloidosis. The disease is always associated to point mutations in the lysozyme gene and fibrils are deposited widely in tissues [56]. The properties of two amyloidogenic lysozyme mutants (I56T and D67H) have been studied in detail and, when compared to those of the wild-type protein, the mutants were found to have reduced structural stability allowing unfolding to take place at least partially at physiologically relevant temperatures [57,58]. Thus, the formation of amyloid fibrils by human lysozyme is likely to occur by the exposure of aggregation-prone region previously hidden in the native structure. The aggregation profile of lysozyme identifies three main "hot spots" corresponding to residues 20–34, 50–62 and 73–104 (Fig. 3). The last large aggregation-prone region includes several local maxima. The first "hot spot" maps in helix B, the second in a β-hairpin of the β-domain and the third includes helix C and a large flanking unstructured region at its N-terminus (Fig. 2). Although there is no experimental characterization of amyloidogenic regions in human lysozyme in the literature, this information is available for the homologous hen lysozyme molecule, which displays an almost identical 3D-structure. The aggregation profile for the hen protein is very similar to that of the human one despite the fact that our input consists solely on the sequence and the identity between both molecules is only of 40%. The equivalent "hot spots" in hen lysozyme comprise residues 24–34, 50–62 and 76–98. Experimental data suggests that the sequence of the β-domain could be of particular relevance for lysozyme aggregation since it unfolds prior to the α-domain [58]. Two peptides encompassing the β-domain of native lysozyme displayed very different behavior: peptide 61–82 appeared to be predominantly unstructured whereas peptide 41–60 showed a high tendency to aggregate and form extended β-sheet structures [59]. The first peptide coincides with a region of very low aggregation propensity in the aggregation profiles, whereas the second one covers the region with the highest aggregation propensity in the profile (residues 50–64). Interestingly enough, a peptide spanning residues 49–64 has been shown to form fibrils with the typical structure of amyloid showing that the first residues of the 41–60 peptide are not relevant for aggregation, as predicted by our approach [60]. Another study has reported that the major fragment incorporated in the core of the fibril structure, as monitored using proteolysis, encompasses the chain region 49–101 [61]. These lysozyme fragments contain helix C and two of the three β-strands of the β-domain of the native protein structure and coincide with the limits of the second and third regions in our predictions (Fig. 2 and 3). This observation could be biologically relevant, since the β-domain and C-helix of the human lysozyme have been shown to unfold locally in the amyloidogenic variant D67H, which is associated with the familial cases of systemic amyloidosis linked to lysozyme deposition [58]. The C-helix is the α-helix with the lowest helical propensity of hen lysozyme according to both theoretical and peptide based studies [59]. This low propensity might be related to the ability of this region to be incorporated into the β-sheet rich fibrillar structured as have been reported for other protein systems [62]. Limited proteolysis of hen lysozyme renders fragments 57–107 and 1–38/108–129 [61]. In the 1–38/108–129 fragment the N-terminal and C-terminal ends of the molecule are joined by a disulfide bond. Only fragment 57–107, but not fragment 1–38/108–129, is able to generate well defined amyloid [61]. Whereas the behavior of the 57–107 fragment is expected from the analysis, one should also expect the fragment 1–38 to have a high tendency to aggregate. Two explanations are possible to account for this discordance. First, it could occur that the helical structure of this region prevents its conversion to β-sheet conformation, since the A-helix displays the highest helical propensity out of all lysozyme helices [59]. The second possibility is that, being joined to the 108–129 region, predicted to have lower aggregation propensity, steric hindrances limit self-assembly or alternatively the average aggregation tendency of this peptide becomes reduced. The analysis supports this last hypothesis reporting a decrease in aggregation propensity (ΔAP = -5.34) in the joined peptide respect the 1–38 peptide alone.

#### Transthyretin

Transthyretin (TTR) is a homotetramer of 127-amino acid subunits. TTR is found in human plasma and cerebral spinal fluid, the plasma form being the amyloidogenetic precursor. TTR constitutes the fibrillar protein found in familial amyloidotic polyneuropathy (FAP) and senile systemic amyloidosis (SSA) [63]. In the case of FAP, the amyloid is associated with a point mutation in the TTR gene. To date, 100 different TTR mutations have been reported, many of which are amyloidogenic [64]. The FAP-associated variants characterized thus far although tetrameric, are destabilized [65]. This destabilization allows tetramer dissociation to the amyloidogenic monomeric intermediate to occur under the influence of mild denaturing denaturation conditions. More than 10 FAP-related variants crystal structures have been solved, revealing that the tertiary and quaternary structures are essentially identical to the wild type form [65]. This observation suggests that the partial denaturation of TTR is a requirement for amyloidogenesis. In this state, the presence of "hot spots" of aggregation could play an especially important role in promoting/driving amyloid formation. According to the analysis of the aggregation profile shown in Fig. 3, the TTR monomer displays three main "hot spots" encompassing residues 10–20, 23–33 and 105–118. Also in this case, aggregation-prone sequences appear to be located in preformed β-sheet structures: A β-strand (11–19), part of the B β-strand (28–36) and G and beginning of H β-strands (104–123) (Fig. 3). Most of these secondary structure elements are involved in the formation of the tetrameric structure: H strands mediate the dimerization whereas A and G provide the contacts for the tetramerization of two preformed dimmers. This explains the protective role played by the TTR quaternary structure against aggregation, since it hides or blocks most of the aggregation prone regions. Dissociation of the tetramer has been reported as a prerequisite for amyloidosis and according to our results might be associated to the exposure of previously hidden amyloidogenic sequences. We detect several short peaks exhibiting high aggregation propensities in the central region (63–94) of TTR. These result from the presence of almost regularly placed residues with low aggregation propensity (Asp, Glu, Arg, Lys, Gly) in this rather hydrophobic sequence, which probably act as disrupters, significantly lowering the aggregation tendency of this particular region, a strategy suggested to be used by nature to avoid edge-to-edge aggregation [66].

To date two different fragments of TTR have been shown to form amyloid fibrils. The peptide 105–115 can be assembled into homogeneous amyloid fibrils with favorable spectroscopic properties [67]. This has allowed to solve its fibrillar structure at high-resolution, showing that it adopts an antiparallel extended beta-strand conformation in the amyloid fibrils [68]. This peptide coincides with the region with the highest aggregation propensity in the profile. Also in excellent agreement with the prediction, the peptide 10–20 is the only other fragment of TTR reported to form amyloid fibrils [69]. No data are available on the region 23–33 but the success of the present method in predicting relevant regions in TTR suggests that it is worth to characterize its *in vitro *aggregation capabilities.

#### Prion protein

Misfolded isoforms of the naturally occurring prion protein (PrP) have been shown to be the causative agents in many mammalian neurodegenerative disorders, including Cruetzfeldt-Jakob disease (CJD) in human, scrapie in sheep, and bovine spongiform encephalopathy in cows. Prion infectivity is unique in that the pathogenic prion form (PrP^Sc^) is involved in the conversion of the endogenous conformation (PrP^C^) into transformed PrP^Sc^. The "protein-only" hypothesis [70] asserts further that no extraneous agents are necessary to explain the unusual behavior of prions. Prion diseases can have infectious, familial, and sporadic origins. The basic infectious mechanism is thought to be a conformational change of the normal prion protein (PrPC) into the pathogenic PrP^Sc ^catalyzed by PrP^Sc ^itself.

The normal prion protein (PrP^C^) is a GPI-anchored glycoprotein constitutively expressed on the surface of primarily neuronal cells. It consists of two structurally different parts; a C-terminal, globular part mainly α-helical in nature (Fig. 2) and an unstructured, N-terminal part [71]. Misfolding of PrP^C ^into PrP^Sc ^occurs posttranslationally and results in increased β-sheet content and gain of protease-resistance. Fig. 3 shows the predicted "hot spots" in the aggregation profile of the full-length human prion protein. They are located at the N-terminus (1–32), in the central region (105–146) and the C-terminus (208–252), respectively.

The role of the detected aggregation-prone sequence at the N-terminus is uncertain since it is out of the protease resistant core of PrP^Sc^. Little information exits about the role of this region, although it appears to be unnecessary both for prion transmission and aggregation. The predicted C-terminal "hot spot" includes almost all the C-terminal α-helix, named C, from the globular domain (Fig. 2). Interestingly, some of the human mutations linked to Creutzfeldt-Jakob disease occur in this region of the prion protein and it has been related to the conversion of PrP^C ^into the toxic PrP^Sc^. Moreover, some strains of PrP resistant to conversion to PrP^Sc ^have been found to bear mutations in helix C, and positions 214 and 218 have been shown to modulate PrP^Sc ^formation [72]. It is also important to note that the main structural differences between prion proteins from different species have been found at the end of helix C [71].

The central region of PrPC linking the unstructured N-terminal part with the globular C-terminal domain is believed to play a pivotal role in the PrP^C ^conformational changes. Extensive studies on the secondary structure and fibrillogenic properties of synthetic peptides of PrP have established that the continuous segment of the prion protein spanning residues 106–147, coincident with the second "hot spot" predicted using our approach, is important for the fibrillogenic properties of the protein [73]. One of the synthetic peptides, that named PrP106-126 within the central region of PrP and near the N-terminal of the protease resistant core of PrP^Sc^, shares many properties with the infectious form as it readily forms amyloid fibrils with a high β-sheet content, shows partial proteinase K resistance and is neurotoxic *in vivo *[74]. The neurotoxicity of PrP106-126 depends on the expression of endogenous PrP^C ^which makes PrP106-126 a relevant model for PrP^Sc ^neurotoxicity [74]. Also another prion derived peptide – PrP118-135 – has been found to cause neuronal death via induction of apoptosis [75]. The toxicity of PrP118-135 is, however, independent of endogenous PrP^C ^expression. Both peptides map in our predicted central aggregation-prone region of PrP^C^.

## Conclusion

Overall, the method described here appears as a useful tool for the identification of protein regions that are especially relevant for protein aggregation and amyloidogenesis both in natively unfolded and properly folded globular proteins (Table 4). The results provide support to the hypothesis that short specific amino acid stretches can act as triggers for the incorporation of polypeptides into amyloid structures. It is interesting to note that in those cases in which structural information allows to delimitate the region incorporated in the core of the fibrillar structure, our predicted "hot spots" and those proved experimentally are considerably shorter than the whole region, suggesting that the role of "hot spots" is to act as specific nucleation points from which the ordered fibrillar structure is expanded.

Nature has provided globular proteins with a reasonable conformational stability in the native state in which, as proved here, aggregation-prone sequences are buried or involved in intra-molecular interactions. This appears as a very successful evolutive strategy to avoid aggregation, since few proteins aggregate from their stable native conformation. Accordingly, amyloid-related mutations in globular proteins usually result in destabilization of the folded state allowing the exposure of previously hidden "hot spots", as those reported here. This explains the scarce success in predicting the effect of mutations in the aggregation of globular proteins (data not shown), whereas the prediction of fatal sequence changes in intrinsically unstructured proteins involved in disease is generally accurate. The effects of such mutations can be explained in most cases by intrinsic factors, as they directly result in changes on the average propensity of the full polypeptide to aggregate.

Besides providing important clues about the mechanism of protein aggregation, this study may be relevant for the therapeutics of amyloid disease, since the identified "hot spots" could be regarded as preferential targets to tackle the deleterious disorders linked to protein deposition. According to our results, different specific strategies should be employed when designing methods to avoid aggregation, depending on the disease being caused by natively unfolded or by globular proteins. In Alzheimer, type II diabetes and Parkinson diseases, shielding the already exposed aggregation-prone regions in the polypeptides by using small compounds or antibodies appears as a promising approach, whereas compounds that will stabilize the native conformation and avoid the exposure of the deleterious "hot spots" will be more effective in the case of globular proteins. Additionally, when gene therapy eventually comes to age, mutations that disrupt aggregation-prone regions in unstructured polypeptides or those which over-stabilize the native state of globular aggregation-prone proteins are expected to be useful approaches to avoid protein deposition and meliorate neurodegenerative and systemic amyloidogenic disorders.

## Methods

### Experimental determination of amino acids aggregation propensities

The CHC of Aβ42 peptide was chosen as a paradigmatic aggregation-prone region for the calculation of the individual effect of each natural amino acid on protein aggregation. The specific effect on Aβ42's deposition promoted by the 20 different natural amino acids when located in the central position of this model "hot spot" were evaluated. Briefly, the wild type Aβ42 gene and its 19 mutants were inserted as a fusion protein upstream of the green fluorescence protein (GFP) and expressed individually in bacteria. In this system, the levels of GFP fluorescence in the cells depend exclusively on the *in vivo *aggregation propensity of the Aβ42 variant [10,25], in such a way that changes in aggregation propensities promoted by the different mutations can be easily monitored by measuring the fluorescence emission of the cells expressing each particular variant and normalizing it relative to that emitted by the cells bearing the wild type sequence. Three independent clones were analyzed for each mutation and each clone was analyzed at least by triplicate to generate consistent data. To obtain the individual aggregation propensities in Table 1, the change promoted by each amino acid was normalized relative to the average change of the pool of 20 amino acids.

### Generation of aggregation profiles and identification of "hot spots"

Different experimental data suggest that the aggregation of Aβ42 occurs from a mostly unfolded conformation in which the CHC is exposed to solvent [76]. Assuming that the individual intrinsic aggregation propensities obtained analyzing this particular protein region will probably apply for any unfolded sequence; an aggregation profile was generated for every protein in this study through a simple assignment of the values in Table 1 to each individual residue in the corresponding sequences. Since "hot spots" are clusters of consecutive residues, the sequence was scanned by using a five residues sliding window. "Hot spots" in the sequence were identified as those protein regions at least five residues in length (the minimal size shown to date to be required for a peptide to form amyloid fibrils similar to those formed by whole polypeptides [77], in which the aggregation propensity is above the average aggregation propensity of the complete sequence. The average propensity of the polypeptide was calculated as the sum of the aggregation propensities of its individual amino acids divided by the number of residues.

### Analysis of the effect of changes in the polypeptide sequence on aggregation

The concept of "hot spot" of aggregation implies that the contribution of a particular residue in a protein sequence on protein aggregation is somehow modulated by its immediate neighbors. According to this, the effects of mutation on protein aggregation can not be properly calculated by a simple subtraction of the intrinsic aggregation propensities of the wild type and mutant residues. Instead, to provide a more general description of the effect of the change on the overall aggregation propensity, the individual aggregation profiles for the wild type protein and the different mutants are obtained and the differences between the areas below the corresponding profiles are calculated. The area between each profile was always normalized by the number of residues in the considered species to compare between the aggregation propensities of the complete protein and fragments coming from proteolysis, chemical synthesis or other processes. The difference between normalized areas, multiplied by a 100 factor, was designed as the change in average aggregation propensity (ΔAP). ΔAP will be positive if the mutation is predicted to increase the aggregation propensity of the polypeptide chain and negative if it is predicted to increase solubility.

## Abbreviations

AD Alzheimer's disease

Aβ Amyloid-β-protein

CHC Central hydrophobic cluster

FAD Familial Alzheimer diseases

FAP Amyloidotic polyneuropathy

GFP Green fluorescent protein

IAPP Islet amyloid polypeptide

NAC Non-Aβ component of amyloid plaques

PrP Prion protein

PrP^Sc ^Pathogenic prion form

SSA Senile systemic amyloidosis

TTR Transthyretin

ΔAP Change in average aggregation propensity

β2-m β2-Microglobulin

## Authors' contributions

NSG and IP performed most of the experiments and prepared the final data and figures. FXA and JV contributed to data interpretation and manuscript redaction. SV directed the work and prepared the manuscript.

## Figures and Tables

**Table 1:** Relative experimental aggregation propensities of the 20 natural amino acids derived from the analysis of mutants in the central position of the CHC in amyloid-β-protein.

Amino acid	
I	1.822
F	1.754
V	1.594
L	1.380
Y	1.159
W	1.037
M	0.910
C	0.604
A	-0.036
T	-0.159
S	-0.294
P	-0.334
G	-0.535
K	-0.931
H	-1.033
Q	-1.231
R	-1.240
N	-1.302
E	-1.412
D	-1.836

**Table 2:** Comparison of predicted and experimental changes in aggregation for Aβ variants.

Mutation	ΔAP*	Observed aggregation^‡^
A21G	-1.22	-
E22G	+2.14	+
E22Q	+0.44	+
F19P	-5.09	-
F19T	-4.73	-
I31L	-1.07	-
I32L	-1.07	-
I41G	-5.76	-
I41A	-4.52	-
I41L	-1.075	-
A42G	-1.21	-
A42V	+3.95	+
Δ1–4	+4.82	+
Δ1–9	+21.86	+
Δ40–42	-8.34	-
Δ41–42	-4.26	-
V12E+V18E+M35T+I41N	-18.96	-
F19S+L34P	-9.18	-

* Change in average aggregation propensity

‡ Changes in aggregation determined experimentally.

**Table 3:** Comparison of predicted and experimental changes in aggregation for IAPP variants, relative to the corresponding human IAPP sequence.

Variant	ΔAP*	Observed aggregation^‡^
(20–29) Cat	-5.12	=
(20–29) Rat	-16.46	-
(20–29) Hamster	-32.73	-
R18H	+0.94	+
L23F	+1.70	+
V26I	+0.42	+
R18H+L23F+V26I	+3.06	+
(22–27) N22A	+31.53	+
(22–27) F23A	-42.96	-
(22–27) G24A	+11.96	+
(22–27) I26A	-44.5872	-
(22–27) L27A	-33.99	-
S20G	-1.09	+
ProIAPP	+31.40	+?

* Change in average aggregation propensity

‡ Changes in aggregation determined experimentally.

? Not yet proved experimentally.

**Table 4:** List of the predicted "hot spots" in the different disease-linked polypeptides in this study and comparison with the available experimental data. Experimental "hot spots" refer to those protein regions shown to be involved in the aggregation process of the corresponding polypeptide. It is also noted if the predicted "hot spot" has been described as a structural element of the amyloid fibrils formed by the different peptides and proteins in the study.

**Protein**	**Predicted "Hot Spots"**	**Experimental "Hot Spots"**	**Regions in the fibrils**
**Amyloid-β-protein**	16–21	+	+
	30–36	+	+
	38–42	+	+
**Islet amyloid polypeptide**	12–18	+	uncertain
	22–28	+	uncertain
	1–18	No experimental data available	uncertain
**α-Synuclein**	27–56	+	uncertain
	61–94	+	+
**β2-Microgobulin**	21–31	+	+
	56–69	+	+
	79–85	+	+
	87–91	+	+
**Lysozyme (hen)**	24–34	-	-
	50–62	+	+
	76–98	+	+
**Transthyretin**	10–20	+	+
	23–33	No experimetal data available	uncertain
	105–118	+	+
**Prion Protein**	1–32	No experimetal data available	uncertain
	105–146	+	+
	208–252	No experimetal data available	uncertain
